# Tocilizumab decreases T cells but not macrophages in the synovium of patients with rheumatoid arthritis while it increases the levels of serum interleukin-6 and RANKL

**DOI:** 10.1136/rmdopen-2021-001662

**Published:** 2021-06-09

**Authors:** Katerina Chatzidionysiou, Alexandra Circiumaru, Bence Rethi, Vijay Joshua, Marianne Engstrom, Aase Hensvold, Erik af Klint, Anca Catrina

**Affiliations:** 1 Rheumatology Unit, Department of Gastroentorogy, Dematology and Rheumatology, Karolinska University Hospital, Stockholm, Sweden; 2 Department of Medicine Solna, Karolinska Institute, Stockholm, Sweden; 3 Centrum for Rheumatology, Academic Specialist Centrum, Stockholm, Sweden

**Keywords:** treatment, arthritis, rheumatoid, synovitis

## Abstract

**Objectives:**

Our knowledge about the effect of tocilizumab (TCZ) on the synovium in rheumatoid arthritis (RA) is limited. The aim of this study was to investigate the effect of TCZ on citrullination and on inflammation in the synovial tissue and in the peripheral blood.

**Methods:**

15 patients with RA underwent synovial biopsy before and 8 weeks after TCZ initiation. Clinical evaluation was performed at baseline and at 8 weeks. Using immunohistochemistry, we evaluated the expression of CD68, CD3, CD20, osteoprotegerin (OPG) and receptor activator for nuclear factor-κB ligand (RANKL) before and after treatment with TCZ. We also analysed the expression of protein arginine deiminase (PAD)-2 and PAD-4 enzymes in the synovial tissue and protein citrullination patterns with the help of anticitrullinated protein antibody (ACPA) clones 1325:04C03 and 1325:01B09. Serum levels of interleukin-6 (IL-6), IL-8, RANKL, OPG and C-terminal crosslinked telopeptide type II collagen were measured by ELISA. Paired-wise Wilcoxon signed-rank test was used to compare median values before and after treatment.

**Results:**

Disease activity in patients was reduced from baseline to 8 weeks. Although PAD-2 and PAD-4 expressions remained unchanged after TCZ treatment, the binding of one ACPA clone decreased in the synovial tissue. TCZ did not affect the number of CD68+ macrophages or CD20+ B cells but induced significant decrease in the number of CD3+ T cells. RANKL and OPG expression remained unchanged in the synovial tissue. A significant increase in the levels of IL-6 and RANKL was observed in the serum. This increase was statistically significant in patients who responded to TCZ (achieving Clinical Disease Activity Index low disease activity or remission) but not in non-responders.

**Conclusions:**

TCZ reduced synovial T-cell counts but not macrophages. A significant increase of serum IL-6 was observed in responders.

**Key messages**

**What is already known about this subject?**

- Tocilizumab (TCZ), a monoclonal antibody against interleukin-6 receptor (IL-6R) is effective in reducing inflammation and inhibiting structural damage in rheumatoid arthritis. There is limited evidence concerning the immunomodulatory effects of TCZ both in the synovium and in the serum.

**What does this study add?**

- IL-6R blockade reduces synovial T-cell counts, especially in patients who respond to therapy, suggesting that T-cell activation is a major target of IL-6R blockade.

**How might this impact on clinical practice or further developments?**

- A significant increase of serum IL-6 was observed in responders, suggesting a direct effect of IL-6R blockade and a possible monitoring tool of treatment effectiveness.

## Introduction

Interleukin-6 (IL-6) is a key cytokine in rheumatoid arthritis (RA). It is secreted from a wide variety of cells including macrophages, T cells, B cells and synovial fibroblasts, and is regarded as upper-rank cytokine in the hierarchical cytokine network involved in the pathogenesis of RA. It has a wide range of functions, such as in B-cell proliferation and antibody production, haematopoiesis and T-cell differentiation.^1 2^ IL-6 triggers the production of acute-phase proteins such as C-reactive protein (CRP) from the liver. In addition, it activates synovial fibroblasts to express matrix metalloproteinases and receptor activator of nuclear factor-κB ligand (RANKL), which induces the differentiation of osteoclasts contributing to bone resorption and bone erosions.^3 4^ Tocilizumab (TCZ) is a humanised monoclonal antibody against IL-6 receptor, approved for the treatment of active RA both as monotherapy and in combination with methotrexate. Its efficacy and acceptable safety profile has been demonstrated in several large randomised controlled trials, leading to its approval from regulatory authorities.^5–8^

The aim of RA treatment is to reduce synovial inflammation and prevent joint destruction. Studying the effects of different antirheumatic therapies on the synovium helps us understand the mechanism of action of the different therapeutic agents, but gives also the opportunity to identify potential predictors of response. Previous studies have shown a profound effect of different treatments, such as glucocorticoids, methotrexate, tumour necrosis factor inhibitors, on synovial cells, such as macrophages, T cells and B cells.^9–11^ Protein modification through post-translational citrullination in the rheumatoid joint is thought to play an important role in perpetuation of local chronic inflammation in the presence of specific anticitrulline immunity. It has previously been shown that antirheumatic treatment can actively modulate synovial citrullination.^12^

There is limited evidence concerning the immunomodulatory effects of TCZ both in the synovium and in the serum. The aim of this study was to characterise the effect of TCZ treatment on citrullination and on inflammation; intra-articular, in the synovial tissue, and extra-articular, in the peripheral blood.

## Methods

### Patient population

Fifteen consecutive patients from Karolinska University Hospital with definite RA, according to American College of Rheumatology 1987 criteria,^13^ independent of disease duration, who failed treatment with at least one conventional synthetic disease modifying antirheumatic drugs (csDMARDs) or biologic disease modifying antirheumatic drug (bDMARDs) and would start treatment with TCZ were included in this study during 2010–2016. Dose of oral glucocorticoids (GCs) and csDMARDs had to be stable at least 4 weeks before entering the study. The demographic and clinical characteristics of the patients at baseline (=start of TCZ treatment) are summarised in table 1. The proportion of bDMARD-naive patients was 27% (4 out of 15 patients).

**Table 1:** Characteristics of the 15 patients with RA at baseline (=the time of initiation of TCZ)

Variable	
Age (years), median (IQR)	65.6 (58.3–79.0)
Sex (% female)	13/15 (93%)
Disease duration (years), median (IQR)	4 (1–13)
RF (% pos)	8/15 (53%)
Anti-CCP (% pos)	9/15 (60%)
Number of prior csDMARDs, median (IQR)	1 (1–2)
Number of prior bDMARDs, median (IQR)	1 (0–2)
Concomitant GCs	8/15 (53%)
Concomitant csDMARDs	4/15 (27%)
DAS28, median (IQR)	5.9 (4.7–6.8)
CDAI, median (IQR)	32.4 (21.2–40.6)
ESR (mm/hour), median (IQR)	34 (15–69)
CRP (mg/L), median (IQR)	11 (5–27)

anti-CCP, antibodies against citrullinated peptides; bDMARDs, biologic disease-modifying antirheumatic drugs; BL, baseline; CDAI, Clinical Disease Activity Index; CRP, C-reactive protein; csDMARDs, conventional synthetic disease-modifying antirheumatic drugs; DAS28, Disease Activity Score based on 28 joint count; ESR, erythrocyte sedimentation rate; GCs, glucocorticoids; RF, rheumatoid factor; SJC, swollen joint count; TCZ, tocilizumab; TJC, tender joint count.

Ultrasound-guided synovial biopsies were obtained from knee (N=15), wrist (N=10) and meta-carpal-phalangeal joints (N=4) before and 8 weeks after initiation of TCZ from 14/15 patients.^14^ One patient discontinued treatment with TCZ and was excluded from the study. Clinical evaluation was performed at baseline and at the time of the second synovial biopsy. Serum samples were also obtained at these two time points.

### Clinical efficacy

Efficacy of treatment was assessed at week 8 by Disease Activity Score based on 28 joint count (DAS28), Clinical Disease Activity Index (CDAI). Patients were categorised as responders if they achieved CDAI low disease activity or remission (CDAI≤10) and non-responders if they exhibited moderate or high disease activity according to CDAI (CDAI>10) at week 8. The reason for using CDAI and not DAS28 was the absence of acute phase reactants in the CDAI score, since it is known that TCZ has a direct effect on both erythrocyte sedimentation rate (ESR) and CRP.

### Synovial biopsy handling and immunohistochemical analyses

Synovial biopsy samples were snap-frozen during ultrasound-guided biopsies in dry-ice cooled isopentane. Serial cryostat sections (7 µm) were fixed for 20 min with formaldehyde and stored at −70°C. The sections were washed with PBS+0.1% saponin and blocked with 1% H_2_O_2_ for 60 min at room temperature (RT) in dark. The sections were then washed, blocking with 20% AB serum before primary antibodies were added to the sections was performed, and the sections were incubated overnight at RT in dark with primary monoclonal antibodies. The following antibodies were used: mouse-antihuman RANKL antibody (12A668), mouse-antihuman osteoprotegerin (OPG) antibody (MAB805) from R&D Systems, Minneapolis, Minnesota, mouse-antihuman CD3 (SK7; BD Biosciences, San Jose, California, USA), mouse-antihuman CD68 (KP1; Dako, Glostrup, Denmark) and mouse-antihuman CD20 (L26, Dako). Matched IgG isotype controls were included for all markers. Presence of citrullinated proteins was detected by using human IgG1 1325:04C03 biotinylated and 1325:01B09 biotinylated anticitrullinated protein antibody (ACPA) clones.^15^ For detection of protein arginine deiminase (PAD) enzyme expression, we used one antimouse monoclonal PAD-4 (ab57167, Abcam) and one rabbit-antihuman monoclonal PAD-2 (ab56928, Cosmo Bio Co Ltd, Japan). After the overnight incubation, the sections were washed and incubated with 1% horse or goat serum for 15 mins in RT. Biotinylated horse antimouse secondary antibody (Vector Laboratories, California, USA) or biotinylated goat-antirabbit were used (for the commercial antibodies) and the sections were incubated for 30 min. Then all sections included C03 and B09 were incubated with the ABC elite (Vector kit) for 45 min in dark and developed with 3,3'-diaminobenzidine (Vector kit) for 7 min. The sections were counter-stained with haematoxylin and analysed using light microscope (Leica Reichert Polyvar 2, magnification 25×). For each biomarker a minimum of six sections were evaluated.

Evaluation of all immunohistochemistry (IHC) variables was performed by two blinded independent observers (KC, ME) using a semiquantitative score on a 0–3 scale (0: no staining; 1: weakly stained; 2: moderate staining; 3: strongly staining). We also performed a global synovitis grading on routine H&E stained slides, according to the three synovial membrane features (synovial lining cell layer, stroma cell density and inflammatory infiltrate), the ranking of alterations being on a scale from none (0), slight (1) and moderate (2) to strong (3), according to Krenn score.^16^ The values of the parameters were summarised and interpreted as follows: 0–1, no synovitis; 2–4, low‐grade synovitis; and 5–9, high‐grade synovitis.

### Serum analyses

After centrifugation of blood samples, sera were stored at −80°C until analysis. ELISA was used to measure the serum levels IL-6, IL-8 (R&D Systems), free soluble RANKL, total OPG (Biomedica, Vienna, Austria) and C-terminal crosslinked telopeptide type II collagen (CTX-II, Aviva Systems Biology, San Diego, CA, USA).

### Statistical analyses

Statistical analysis was performed by using the Wilcoxon test for comparison of paired samples, the Mann-Whitney test for comparison of independent samples, and the Spearman rank correlation test. Differences between proportions were analysed with the Fisher’s exact test. P values <0.05 were considered statistically significant. Stratified analyses were performed based on clinical response (responders vs non-responders, as described above) and on seropositivity (RF and/or ACPA positive vs double-negative). All analyses were performed by SPSS (IBM Corp, IBM SPSS Statistics for Windows, V.25.0, Armonk, New York, USA).

## Results

### Clinical efficacy and immunohistological changes

Disease activity was prospectively evaluated in the 14 patients with RA who remained on treatment (one patient was excluded because of TCZ discontinuation) at baseline and 8 weeks after the initiation of TCZ therapy. Disease activity was improved in all patients but one. As expected, a highly significant reduction of acute phase reactants, ESR and CRP, were observed from baseline to 8 weeks. Significant reductions were observed for CDAI and its components (table 2). Out of 14 patients, 10 of them were categorised as responders (CDAI low disease activity or remission) and 4 of them as non-responders (CDAI moderate or high disease activity). No differences were observed between ACPA positive and negative patients.

**Table 2:** Clinical efficacy of TCZ treatment. All values represent median (

	Baseline	8 weeks	Wilcoxon signed-rank test, p value
DAS28	5.9 (4.7–6.8)	2.98 (2.0–3.8)	0.028
CDAI	32.4 (21.2–40.6)	6.7 (3.6–12.1)	0.005
SJC	9 (3–14)	1 (0–4.25)	0.009
TJC	10 (4–15)	1 (0–2.25)	0.017
ESR	34 (15–69)	6 (5–16)	0.001
CRP	11 (5–27)	1 (1–2)	0.005

All values represent median (IQR).

BL, baseline; CDAI, Clinical Disease Activity Index; CRP, C-reactive protein; DAS28, Disease Activity Score based on 28 joint count; ESR, erythrocyte sedimentation rate; SJC, swollen joint count; TJC, tender joint count.

A significant reduction in the global synovitis score was observed (median (IQR): 5 (4–6)–1 (0–4), p=0.001). This significant reduction was observed in the responders’ group (median (IQR): 5 (2.75–6.25)–1 (0–2.5), p=0.007). In non-responders, a numerical but not statistically significant reduction was observed (median (IQR): 6 (4.5–8.25)–2.5 (0.25–5.5), p=0.07).

**Figure 1:**
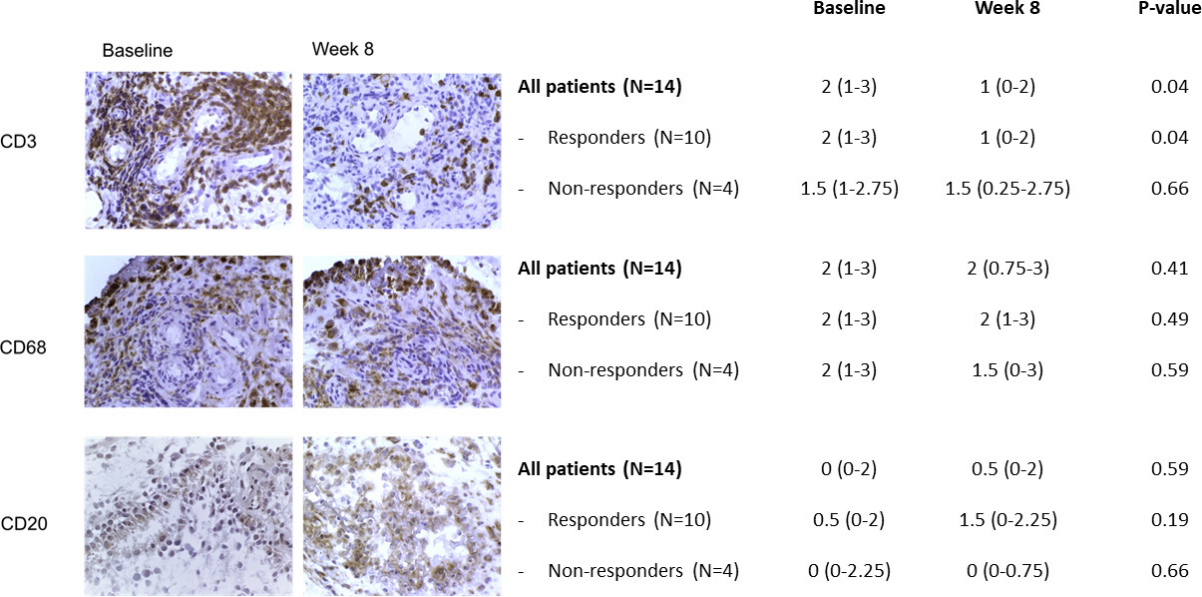
Effect of tocilizumab on cell populations in synovial biopsy samples obtained from patients with active rheumatoid arthritis prior to and 8 weeks after initiation of tocilizumab. Immunohistologic findings for T cells (CD3), macrophages (CD68) and B cells (CD20) in representative synovial tissue samples are shown. Results of semiquantitative evaluations were obtained in 14 paired samples (obtained at baseline and week 8). Values are shown as median (IQR) in the table for all patients and stratified according to clinical response (Clinical Disease Activity Index remission/low disease activity vs Clinical Disease Activity Index moderate/high disease activity at week 8). The changes in median score from baseline to week 8 were compared by Wilcoxon’s matched pairs signed-rank test.

We observed a significant decrease in the number of synovial T cells, as evaluated by CD3 staining (figure 1). After stratification according to the clinical response, this significant effect of treatment on synovial T cells was present only in the responders' group. No significant reduction of macrophages or B cells was observed, neither in the whole group nor in the responders/non-responders (figure 1).

### Effects of TCZ on synovial protein citrullination

**Figure 2:**
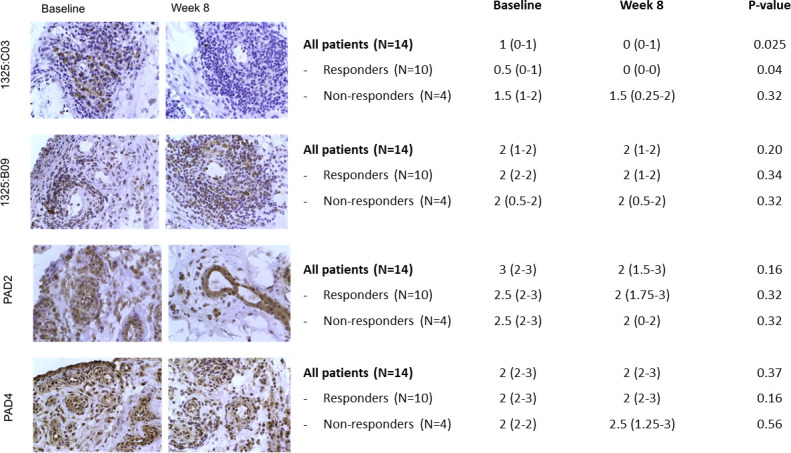
Effect of tocilizumab on citrullination in synovial biopsy samples obtained from patients with active rheumatoid arthritis prior to and 8 weeks after initiation of tocilizumab, as assessed by expression of citrullinated peptides (1325:hIgG C03 and 1325:B09) and PAD-2 and PAD-4 enzymes. Immunohistologic findings in representative synovial tissue samples are shown. Results of semiquantitative evaluations were obtained in 15 paired samples (obtained at baseline and week 8). Values are shown as median (IQR) in the table for all patients and stratified according to clinical response (Clinical Disease Activity Index remission/low disease activity vs Clinical Disease Activity Index moderate/high disease activity at week 8). The changes in median score from baseline to week 8 were compared by Wilcoxon’s matched pairs signed-rank test. PAD, protein arginine deiminase.

A significant reduction was observed in the binding of one ACPA clone (1325:04C03) to the synovial tissue after TCZ treatment, especially in patients achieving good response, whereas binding of the other tested ACPA cloned (1325:01B09) remained unaffected (figure 2). No significant changes were observed in the expression of the PAD-2 and PAD4 enzymes (figure 2). After stratification based on seropositivity no differences were observed between seropositive and seronegative patients.

### Immunomodulatory effects of TCZ on proinflammatory serum cytokines and chemokines

TCZ treatment significantly increased IL-6 and soluble free RANKL serum levels, while serum levels of IL-8 and OPG remained unchanged (table 3). This increase was statistically significant in patients who responded to TCZ (achieving CDAI low disease activity or remission) but not in non-responders (table 3), and in patients on concomitant GCs (median (IQR) serum level=0.11 (0.07–0.32)–0.21 (0.18–0.39), p=0.01) but not in those without (median (IQR) serum level=0.15 (0.03–0.25)–0.16 (0.11–0.59), p=0.23).

**Table 3:** Median (IQR) values of IL-6, IL-8, CTX-II, RANKL and OPG serum levels at baseline and 8 weeks after initiation of TCZ treatment

	Baseline	Week 8	P value
IL-6 (pg/mL)			
Total	9.1 (1.1–14.8)	34.2 (25.4–51.9)	0.001
Responders	3.6 (1.2–10.7)	33.7 (20.2–47.8)	0.005
Non-responders	16.9 (13.4–20.8)	34.2 (32.0–48.3)	0.068
IL-8 (pg/mL)			
Total	15.3 (11.5–20.1)	15.3 (12.8–21.5)	0.638
Responders	14.6 (10.2–20.1)	15.8 (11.2–21.5)	0.445
Non-responders	19.1 (14.2–23.2)	15.3 (13.6–35.5)	1.000
RANKL (pmol/mL)			
Total	0.11 (0.05–0.25)	0.20 (0.15–0.38)	0.013
Responders	0.09 (0.05–0.27)	0.21 (0.18–0.38)	0.008
Non-responders	0.18 (0.05–0.28)	0.15 (0.08–0.69)	0.465
OPG (pmol/L)			
Total	7.04 (5.04–9.79)	7.20 (5.28–8.65)	0.272
Responders	7.33 (5.04–10.25)	7.20 (5.28–8.65)	0.285
Non-responders	6.62 (4.79–9.14)	6.39 (4.57–8.89)	1.000
CTX-II (ng/mL)			
Total	1525.1(1178.4–1672.6)	1715.1 (1393.1–2660.1)	0.433
Responders	1518.0(1172.3–1647.9)	1782.54(1310.6–2668.3)	0.169
Non-responders	1598.8(1369.9–3242.6)	1579.18(1420.0–2600.2)	0.465

CTXII, C-terminal crosslinked telopeptide type II collagen; IL-6, interleukin-6; IL-8, interleukin-8; OPG, osteoprotegerin; RANKL, receptor activator for nuclear factor-κB ligand.

**Figure 3:**
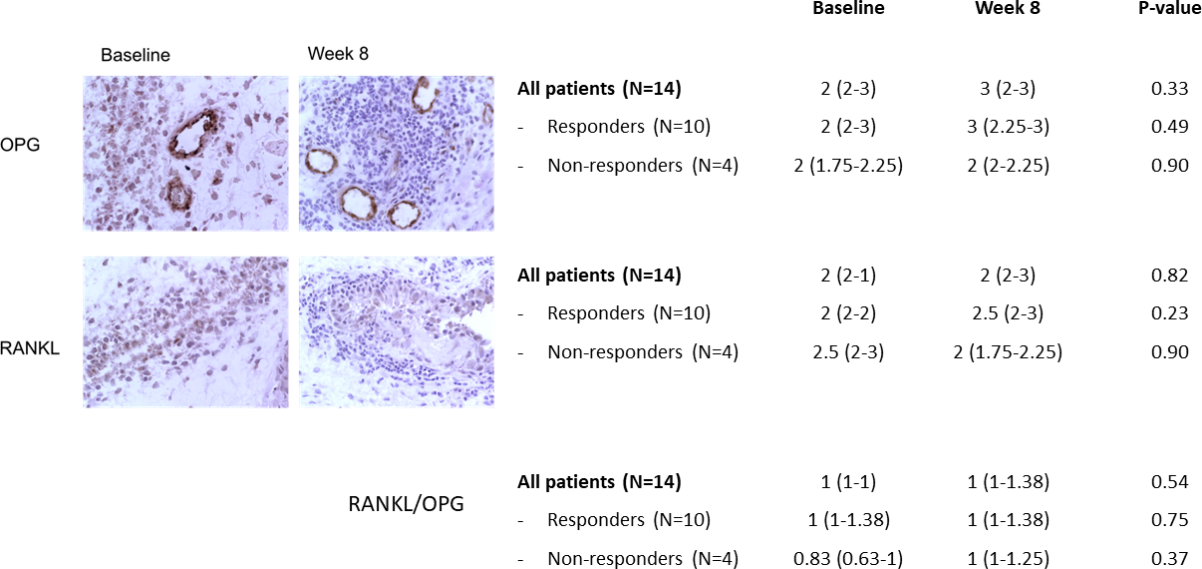
Effect of tocilizumab on expression of RANKL and OPG in synovial biopsy samples obtained from patients with active rheumatoid arthritis prior to and 8 weeks after initiation of tocilizumab. Immunohistologic findings in representative synovial tissue samples are shown. Results of semiquantitative evaluations were obtained in 15 paired samples (obtained at baseline and week 8). Values are shown as median (IQR) in the table for all patients and stratified according to clinical response (Clinical Disease Activity Index remission/low disease activity vs Clinical Disease Activity Index moderate/high disease activity at week 8). The changes in median score from baseline to week 8 were compared by Wilcoxon’s matched pairs signed-rank test. OPG, osteoprotegerin; RANKL, receptor activator for nuclear factor-κB ligand.

We have also analysed RANKL and OPG expression in the synovial tissues using IHC, where we did not detect a difference in response to TCZ treatment (figure 3). Notably, in spite of the increase of circulating RANKL levels, the bone resorption marker CTX-II was not altered significantly in response to TCZ therapy (table 3).

## Discussion

The exact molecular mechanism of action for TCZ in synovium and peripheral blood is not fully understood, although its effectiveness is well established. In this study, we could show that treatment with TCZ significantly reduced the grade of synovial inflammation and the number of synovial tissue T cells. This was observed in the responders but not in non-responders and in both ACPA-positive and negative patients. Ducreux *et al* have previously described a significant decrease in the expression of T-cell activation genes in the RA synovium, as well as a significant decrease in synovial T cells.^17^ It is interesting to note that IL-6 was originally described as T-cell activation factor.^18^ Our data further support the hypothesis that T-cell activation is a major target of IL-6 receptor (IL-6R) blockade, something that could have potential prognostic value of TCZ treatment in RA. Patients who responded to treatment had a higher expression of T cells at baseline, although not statistically significant. The lack of statistical significance could be due to lack of true difference, but could also be due to the lack of power, so the risk of type II error might exist.

Unexpectedly, no significant reduction in macrophages was observed. In the study by Ducreux *et al*, a significant reduction of CD68+ cells was observed, in contrast to our study, although the reduction was not as striking as of CD3+ T cells.^17^ CD68+ macrophages in the sublining layer in synovial tissue have been shown to be one of the best activity markers for RA and the optimal indicator of effective therapy.^19–21^ There is evidence that there are different macrophage subpopulations in the human synovium with the potential to contribute either to joint homeostasis or to chronic inflammation in RA.^22 23^ Discrimination of these two different populations would be of importance, and could potentially explain the lack of significant reduction in the total population of macrophages in our study. Another possible explanation about the lack of significant effect of the treatment on macrophages could be the timing of the second synovial biopsy. We usually evaluate the clinical effect of a bDMARD after 3 months of therapy. However, it is well-known that the majority of the bDMARDs, with the exception of rituximab and abatacept, are associated with rapid improvement, both on a clinical and on a molecular level. Infliximab has been shown to reduce T cells in synovial biopsies taken 4 weeks after treatment.^24^ In another study of infliximab versus placebo in which 24 patients with active RA underwent arthroscopy and biopsy before, and 48 hours after infliximab, revealed a significant reduction in CD68 intimal macrophages, as well as a non-statistically significant reduction in CD68 macrophages, T cells and plasma cells in the sublining.^11^ Taking into consideration the results of the studies above, it is less likely that the 8 weeks time-point is too early to observe a significant reduction of macrophages.

ACPAs comprise a collection of antibodies with different specificities towards citrullinated (cit)-epitopes. Although some level of cross-reactivity has been described for the monoclonal ACPAs used in the present study, both are characterised by several unique reactivities (eg, cit-vimentin at regions 2–17 or 60–75 for 1325:04C03 and cit-fibrinogen at regions 36–52 or 563–583 for 1325:01B09).^15^ It was previously shown that 1325:04C03 could bind to osteoclast precursor cells and enhance osteoclastogenesis and bone resorption,^25^ whereas 1325:01B09 reacted with synovial fibroblasts.^26^ In our study, we could observe a significant reduction in the binding of 1325:04C03 to the synovium after TCZ treatment, whereas 1325:01B09 binding remained unchanged. TCZ therapy could therefore alter the composition of citrullinated autoantigens and this might subsequently influence how ACPAs react with the different synovial cell types. Interestingly we did not see change in RANKL mirroring and disturbance in the osteoclast/osteoblast homeostasis.

The quantitative analysis of the levels of IL-6 in serum of patients before and after treatment revealed a significant increase of the levels of IL-6 in patients who responded to treatment with TCZ. Similar results with increase in IL-6 were observed in previous studies.^27 28^ The mechanism(s) behind this effect is not known. TCZ is a competitive inhibitor of soluble as well as membrane bound IL-6 receptors. The cellular effects of IL-6 are mediated through the IL-6R and thus blocking the receptor hinders IL-6 from exerting its proinflammatory effects. Increased serum IL-6 after TCZ administration might be caused by inhibition of IL-6R-mediated clearance, and this free IL-6 cannot induce intracellular signals because IL-6R is occupied by TCZ.

An unexpected but unique finding was the significant increase of free soluble RANKL levels in the sera of TCZ-treated individuals, especially in the group of responders. There are no other studies on RANKL changes after TCZ treatment. Previous studies have shown reduction in the levels of RANKL in serum of patients with RA with various DMARDs.^29^ In our study, this significant increase in RANKL was observed in the subgroup of patients treated with GCs. However, the small number of patients does not allow us to draw any safe conclusions from the subgroup analysis. IL-6 is a key molecule in driving osteoclastogenesis and bone resorption. RANKL is induced by IL-6 in mesenchymal cells, which promotes osteoclast activation, and IL-6 also influences T lymphocytes to support osteoclastogenesis.^30^ Previous data have shown that the complex of IL-6 with IL-6R is effective in inducing osteoclast formation, but not IL-6 or IL-6R alone.^31^ In an older study by Axmann *et al*, IL-6R blockade inhibited inflammatory bone erosion through direct interference with osteoclast formation independently of its anti-inflammatory activity.^32^ This has been confirmed in TCZ-treated patients with RA.^33^ The increase seen in RANKL levels after treatment could imply a RANKL-independent mechanism of inhibition of structural damage in RA. This however remains a speculation. At the same time, the OPG and the OPG-bound RANKL are in high excess compared with the free RANKL, which means that even a small change in OPG concentration would have significant impact in the RANKL.

Three distinct histomorphological patterns of RA synovitis have been described: follicular, diffuse and pauci-immune. Immunophenotypic characterisation of these three histomorphological patterns demonstrates B cells principally in ‘follicular’ synovitis but absent/low in the ‘pauci-immune’ and ‘diffuse’ pathotypes, the latter predominated by CD68+ monocytes.^34^ In this study, we tried to categorise patients in the ‘follicular’ pattern (N=8) versus the two other patterns combined (N=6) at baseline, but we could not observe any correlation to treatment response (data not shown). Interestingly, in the first-ever biopsy-based randomised controlled trial in RA, in patients histologically classified as B-cell poor, there was no statistically significant difference in the primary endpoint (50% improvement in CDAI50% from baseline) between the rituximab-treated group and the TCZ group. However, in the synovial biopsies classified as B-cell poor with RNA sequencing the TCZ group had a significantly higher response rate compared with the rituximab group for CDAI50% (rituximab group 12 (36%) of 33 patients vs TCZ group 20 (63%) of 32 patients; difference 26% (2 to 50), p=0·035).^35^ Since the synovium is the ultimate target of RA, it is very likely that we could identify potential treatment biomarkers. Indeed, in the study mentioned above, in patients with RA with low or absent B-cell expression in the synovium, TCZ seems to be more effective than rituximab, a B-cell depleting agent. The absence of a significant biomarker associated to TCZ treatment response in our study could be due to the limited number of patients recruited.

The biggest limitation of this study is the small patient population and the heterogeneity with regard to disease duration and prior treatments. On the other hand this is one of the few studies on the effects of TCZ on synovial citrullination and inflammation with paired samples from synovium. It is based on a real-life patient population receiving TCZ treatment in clinical practice. Validation of these results in other cohorts is needed.

## Conclusions

IL-6R blockade reduces synovial T-cell counts, especially in patients who respond to therapy, suggesting that T-cell activation is a major target of IL-6R blockade. Interestingly, no effect on the number of macrophages was found. A significant increase of serum IL-6 was observed in responders, suggesting a direct effect of IL-6R blockade and a possible monitoring tool of treatment effectiveness. IL-6R blockade leads to significant decrease in specific antigen citrullination but not overall change in citrullination-mediated enzymes or all antigens.

## Data Availability

All data relevant to the study are included in the article or uploaded as supplementary information.

## References

[1] Nishimoto N, Kishimoto T. Interleukin 6: from bench to bedside. Nat Clin Pract Rheumatol 2006;2:619–26. 10.1038/ncprheum0338 17075601

[2] Gabay C. Interleukin-6 and chronic inflammation. Arthritis Res Ther 2006;8 Suppl 2:S3. 10.1186/ar1917 PMC3226076 16899107

[3] Rannou F, François M, Corvol M-T, et al. Cartilage breakdown in rheumatoid arthritis. Joint Bone Spine 2006;73:29–36. 10.1016/j.jbspin.2004.12.013 16087381

[4] Schett G, Gravallese E. Bone erosion in rheumatoid arthritis: mechanisms, diagnosis and treatment. Nat Rev Rheumatol 2012;8:656–64. 10.1038/nrrheum.2012.153 23007741 PMC4096779

[5] Maini RN, Taylor PC, Szechinski J, et al. Double-blind randomized controlled clinical trial of the interleukin-6 receptor antagonist, tocilizumab, in European patients with rheumatoid arthritis who had an incomplete response to methotrexate. Arthritis Rheum 2006;54:2817–29. 10.1002/art.22033 16947782

[6] Smolen JS, Beaulieu A, Rubbert-Roth A, et al. Effect of interleukin-6 receptor inhibition with tocilizumab in patients with rheumatoid arthritis (option study): a double-blind, placebo-controlled, randomised trial. Lancet 2008;371:987–97. 10.1016/S0140-6736(08)60453-5 18358926

[7] Genovese MC, McKay JD, Nasonov EL. Interleukin-6 receptor inhibition with tocilizumab reduces disease activity in rheumatoid arthritis with inadequate response to disease-modifying antirheumatic drugs: the tocilizumab in combination with traditional disease-modifying antirheumatic drug the. Arthritis Rheum 2008. 10.1002/art.23940 18821691

[8] Burmester GR, Rubbert-Roth A, Cantagrel A. A randomised, double-blind, parallel-group study of the safety and efficacy of subcutaneous tocilizumab versus intravenous tocilizumab in combination with traditional disease-modifying antirheumatic drugs in patients with moderate to severe rheumatoid art. Ann Rheum Dis 2014. 10.1136/annrheumdis-2013-203523 PMC3888614 23904473

[9] Gerlag DM, Haringman JJ, Smeets TJM, et al. Effects of oral prednisolone on biomarkers in synovial tissue and clinical improvement in rheumatoid arthritis. Arthritis Rheum 2004;50:3783–91. 10.1002/art.20664 15593225

[10] Dolhain RJ, Tak PP, Dijkmans BA, et al. Methotrexate reduces inflammatory cell numbers, expression of monokines and of adhesion molecules in synovial tissue of patients with rheumatoid arthritis. Br J Rheumatol 1998;37:502–8. 10.1093/rheumatology/37.5.502 9651076

[11] Smeets TJM, Kraan MC, Van Loon ME. Tumor necrosis factor α blockade reduces the synovial cell infiltrate early after initiation of treatment, but apparently not by induction of apoptosis in synovial tissue. Arthritis Rheum 2003. 10.1002/art.11098 12905468

[12] Makrygiannakis D, Revu S, Neregard P. Glucocorticoids decrease the expression of synovial citrullinated proteins in rheumatoid arthritis. Arthritis Rheum 2009.

[13] Arnett FC, Edworthy SM, Bloch DA. The American rheumatism association 1987 revised criteria for the classification of rheumatoid arthritis. Arthritis Rheum 1988. 10.1002/art.1780310302 3358796

[14] Kelly S, Humby F, Filer A, et al. Ultrasound-Guided synovial biopsy: a safe, well-tolerated and reliable technique for obtaining high-quality synovial tissue from both large and small joints in early arthritis patients. Ann Rheum Dis 2015;74:611–7. 10.1136/annrheumdis-2013-204603 24336336

[15] Steen J, Forsström B, Sahlström P. Recognition of amino acid motifs, rather than specific proteins, by human plasma Cell–Derived monoclonal antibodies to posttranslationally modified proteins in rheumatoid arthritis. Arthritis Rheumatol 2019. 10.1002/art.40699 PMC6563427 30152202

[16] Krenn V, Morawietz L, Burmester G-R, et al. Synovitis score: discrimination between chronic low-grade and high-grade synovitis. Histopathology 2006;49:358–64. 10.1111/j.1365-2559.2006.02508.x 16978198

[17] Ducreux J, Durez P, Galant C, et al. Global molecular effects of tocilizumab therapy in rheumatoid arthritis synovium. Arthritis Rheumatol 2014;66:15–23. 10.1002/art.38202 24449571

[18] Houssiau FA, Coulie PG, Olive D, et al. Synergistic activation of human T cells by interleukin 1 and interleukin 6. Eur J Immunol 1988;18:653–6. 10.1002/eji.1830180427 3130269

[19] Bresnihan B, Pontifex E, Thurlings RM, et al. Synovial tissue sublining CD68 expression is a biomarker of therapeutic response in rheumatoid arthritis clinical trials: consistency across centers. J Rheumatol 2009;36:1800–2. 10.3899/jrheum.090348 19671815

[20] Haringman JJ, Gerlag DM, Zwinderman AH, et al. Synovial tissue macrophages: a sensitive biomarker for response to treatment in patients with rheumatoid arthritis. Ann Rheum Dis 2005;64:834–8. 10.1136/ard.2004.029751 15576415 PMC1755544

[21] Tak PP, Smeets TJM, Daha MR. Analysis of the synovial cell infiltrate in early rheumatoid synovial tissue in relation to local disease activity. Arthritis Rheum 1997. 10.1002/art.1780400206 9041933

[22] Kurowska-Stolarska M, Alivernini S. Synovial tissue macrophages: friend or foe? RMD Open 2017;3:e000527. 10.1136/rmdopen-2017-000527 29299338 PMC5729306

[23] Alivernini S, MacDonald L, Elmesmari A, et al. Distinct synovial tissue macrophage subsets regulate inflammation and remission in rheumatoid arthritis. Nat Med 2020;26:1295–306. 10.1038/s41591-020-0939-8 32601335

[24] Tak PP, Taylor PC, Breedveld FC. Decrease in cellularity and expression of adhesion molecules by anti- tumor necrosis factor α monoclonal antibody treatment in patients with rheumatoid arthritis. Arthritis Rheum 1996. 10.1002/art.1780390702 8670314

[25] Krishnamurthy A, Joshua V, Haj Hensvold A, et al. Identification of a novel chemokine-dependent molecular mechanism underlying rheumatoid arthritis-associated autoantibody-mediated bone loss. Ann Rheum Dis 2016;75:721–9. 10.1136/annrheumdis-2015-208093 26612338 PMC4819614

[26] Sun M, Rethi B, Krishnamurthy A, et al. Anticitrullinated protein antibodies facilitate migration of synovial tissue-derived fibroblasts. Ann Rheum Dis 2019;78:1621–31. 10.1136/annrheumdis-2018-214967 31481351 PMC6900251

[27] Nishimoto N, Terao K, Mima T, et al. Mechanisms and pathologic significances in increase in serum interleukin-6 (IL-6) and soluble IL-6 receptor after administration of an anti-IL-6 receptor antibody, tocilizumab, in patients with rheumatoid arthritis and Castleman disease. Blood 2008;112:3959–64. 10.1182/blood-2008-05-155846 18784373

[28] Shimamoto K, Ito T, Ozaki Y, et al. Serum interleukin 6 before and after therapy with tocilizumab is a principal biomarker in patients with rheumatoid arthritis. J Rheumatol 2013;40:1074–81. 10.3899/jrheum.121389 23637318

[29] Hensvold AH, Joshua V, Li W, et al. Serum RANKL levels associate with anti- citrullinated protein antibodies in early untreated rheumatoid arthritis and are modulated following methotrexate. Arthritis Res Ther 2015;17. 10.1186/s13075-015-0760-9 PMC4559929 26337028

[30] Palmqvist P, Persson E, Conaway HH, et al. Il-6, leukemia inhibitory factor, and oncostatin M stimulate bone resorption and regulate the expression of receptor activator of NF-kappa B ligand, osteoprotegerin, and receptor activator of NF-kappa B in mouse calvariae. J Immunol 2002;169:3353–62. 10.4049/jimmunol.169.6.3353 12218157

[31] Tamura T, Udagawa N, Takahashi N, et al. Soluble interleukin-6 receptor triggers osteoclast formation by interleukin 6. Proc Natl Acad Sci U S A 1993;90:11924–8. 10.1073/pnas.90.24.11924 8265649 PMC48097

[32] Axmann R, Böhm C, Krönke G, et al. Inhibition of interleukin-6 receptor directly blocks osteoclast formation in vitro and in vivo. Arthritis Rheum 2009;60:2747–56. 10.1002/art.24781 19714627

[33] Smolen JS, Avila JCM, Aletaha D. Tocilizumab inhibits progression of joint damage in rheumatoid arthritis irrespective of its anti-inflammatory effects: disassociation of the link between inflammation and destruction. Ann Rheum Dis 2012;71:687–93. 10.1136/annrheumdis-2011-200395 22121130 PMC3329225

[34] Pitzalis C, Kelly S, Humby F. New learnings on the pathophysiology of RA from synovial biopsies. Curr Opin Rheumatol 2013;25:334–44. 10.1097/BOR.0b013e32835fd8eb 23492740

[35] Humby F, Durez P, Buch MH, et al. Rituximab versus tocilizumab in anti-TNF inadequate Responder patients with rheumatoid arthritis (R4RA): 16-week outcomes of a stratified, biopsy-driven, multicentre, open-label, phase 4 randomised controlled trial. Lancet 2021;397:305–17. 10.1016/S0140-6736(20)32341-2 33485455 PMC7829614

